# Reduced high-density lipoprotein antioxidant function in patients with coronary artery disease and acute coronary syndrome

**DOI:** 10.1172/jci.insight.187889

**Published:** 2025-03-24

**Authors:** Benjamin Sasko, Linda Scharow, Rhea Mueller, Monique Jaensch, Werner Dammermann, Felix S. Seibert, Philipp Hillmeister, Ivo Buschmann, Martin Christ, Oliver Ritter, Nazha Hamdani, Christian Ukena, Timm H. Westhoff, Theodoros Kelesidis, Nikolaos Pagonas

**Affiliations:** 1 Ruhr-University of Bochum, Medical Department II, Marien Hospital Herne, Bochum, Germany.; 2 Department of Cardiology, University Medical Center Brandenburg an der Havel, Medical School Theodor Fontane, Brandenburg an der Havel, Germany.; 3 Department of Cardiology, University Hospital Ruppin-Brandenburg, Medical School Theodor Fontane, Neuruppin, Germany.; 4 Faculty of Health Sciences, Joint Faculty of the Brandenburg University of Technology Cottbus – Senftenberg, the (MHB) Theodor Fontane and the University of Potsdam, Germany.; 5 Center for Internal Medicine II, University Medical Center Brandenburg an der Havel, Medical School Theodor Fontane, Brandenburg an der Havel, Germany.; 6 Medical Department I, Marien Hospital Herne, Ruhr-University of Bochum, Bochum, Germany.; 7 Department of Angiology, Brandenburg Medical School Theodor Fontane, Brandenburg an der Havel, Germany.; 8 Department of Cardiology, Knappschaftskrankenhaus Bottrop, Academic Teaching Hospital, University Duisburg-Essen, Germany.; 9 Department of Cellular and Translational Physiology, Institute of Physiology, and; 10 Institut für Forschung und Lehre (IFL), Molecular and Experimental Cardiology, Ruhr-University Bochum, Bochum, Germany.; 11 Department of Medicine, Division of Infectious Diseases and Geographic Medicine, University of Texas Southwestern Medical Center, Dallas, Texas, USA.

**Keywords:** Cardiology, Inflammation, Metabolism, Atherosclerosis, Cholesterol, Lipoproteins

## Abstract

RESULTS. Participants with CAD (*n* = 723) had 12% higher mean relative levels of nHDL_ox_ compared with those with invasively excluded CAD (*n* = 502, *P* < 0.001). Patients presenting with symptoms of an ACS had the highest nHDL_ox_ values when compared with the elective cohort (median 1.35, IQR 0.97 to 1.85, *P* < 0.001). In multivariate analysis adjusted for age, sex, body mass index, and hypertension, nHDL_ox_ was a strong independent predictor of ACS (*P* < 0.001) but not of CAD (*P* > 0.05).CONCLUSION. HDL antioxidant function is reduced in patients with CAD. nHDL_ox_ is strongly associated with ACS.

TRIAL REGISTRATION. German Clinical Trials Register DRKS00014037.

FUNDING. Brandenburg Medical School Theodor Fontane, the BIOX Stiftung, and NIH grants R01AG059501 and R03AG059462.

BACKGROUND. High-density lipoprotein (HDL) function rather than its concentration plays an important role in the pathogenesis of coronary artery disease (CAD). The aim of the present study was to determine whether reduced antioxidant function of HDL is associated with the presence of a stable CAD or acute coronary syndrome (ACS).METHODS. HDL function was measured in 2 cohorts: 1225 patients admitted electively for coronary angiography and 196 patients with ACS. A validated cell-free biochemical assay was used to determine reduced HDL antioxidant function, as assessed by increased HDL-lipid peroxide content (HDL_ox_), which was normalized by HDL-C levels and the mean value of a pooled serum control from healthy participants (nHDL_ox_; unitless). Results are expressed as median with interquartile range (IQR).

## Abstract 2

<div> <div> <div> Oxidized HDL is linked to increased risk of acute myocardial infarction, highlighting its role in cardiovascular disease pathogenesis due to inflammation and oxidative stress. </div> </div> <cib-overlay> </cib-overlay></div>

## Graphical abstract

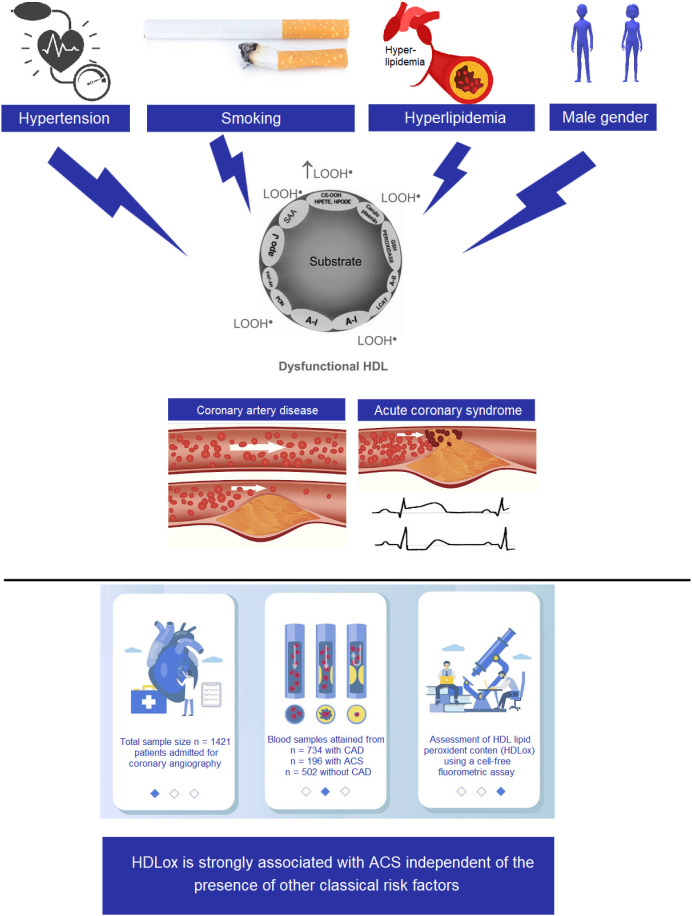

## Introduction

Atherosclerotic cardiovascular disease (CVD) is a major cause of morbidity and mortality worldwide. Coronary artery disease (CAD) contributes to increased mortality and hospitalization in the general population. However, the exact mechanisms that contribute to the pathogenesis of CAD remain unclear. High-density lipoprotein cholesterol (HDL-C) is generally regarded as protective against the development and the progression of CVD and levels of HDL-C constitute an important indicator of CVD events. However, HDL function rather than HDL-C levels may be a more accurate indicator of CVD risk (1, 2). Prior studies confirm that CVD is strongly inversely associated with HDL function. Major antiatherosclerotic roles of HDL include macrophage cholesterol efflux (3), protective endothelial function (4, 5), antiinflammatory effects (6–8), and antioxidant effects (9–11).

In the setting of inflammation, however, HDL becomes functionally impaired, thus elevating CVD risk (12–14). Independently of HDL levels, inflammation affects HDL by decreasing its antiinflammatory and antioxidant activity, increasing associated proinflammatory proteins, lipid hydroperoxide content, and redox activity (HDL_ox_) (15, 16). Furthermore, functionally impaired HDL reduces cholesterol efflux potential and diminishes the ability of HDL to inhibit low-density lipoprotein (LDL) oxidation (12, 14, 15, 17). Thus, given that the protective functions of HDL attenuate pro-oxidant, proinflammatory, and prothrombotic mechanisms (18, 19), the impaired HDL may be a common instigator for development and progression of CAD. This hypothesis is supported by prior studies that have shown an association of dysfunctional HDL with several CVDs, such as CAD (17), ischemic cardiomyopathy (20), or heart failure (21, 22). Furthermore, in the setting of acute cardiovascular events like acute coronary syndrome (ACS) or ischemic stroke, increased oxidative stress has been associated with changes in HDL function and consequently with mortality or adverse outcomes (23–28). HDL oxidation may contribute to the formation of dysfunctional HDL (29, 30) and as previously shown, the oxidative properties of HDL are closely associated with HDL function (31).

Emerging studies have led to growing interest in the assessment of HDL functional properties. However, due to the complexity of the HDL particles, the measurement of HDL function has been difficult to study in humans (25). Currently, HDL functional properties are most often determined by cell-based assays, including the measurement of cholesterol efflux capacity or HDL antiinflammatory capacity (16, 28, 32, 33). However, cell-based assays and the currently available cell-free assays have several limitations, including the lack of standardization and significant heterogeneity regarding types of cells and the type of readout, as reported previously (34, 35). In sum, the use of these assays is complex and time-consuming. Furthermore, several limitations render these cell-based assays inaccessible to many researchers, thus making it difficult to scale up for large-scale clinical trials and/or routine clinical use (34). Cell-free assays may give more robust HDL functional measurements than cell-based assays (36, 37). We have developed a cell-free, reproducible, and rapid fluorometric method that measures HDL-associated lipid peroxide content (HDL_ox_) as a surrogate measure of reduced HDL antioxidant function (36, 38).

Notably, we have shown before that HDL_ox_ contributes directly to atherogenesis in an ex vivo mechanistic assay of atherogenesis (36). Importantly, we have further validated this cell-free fluorometric method of HDL function against other measures of HDL function, including cell-based assays of antiinflammatory function of HDL (31, 36), cholesterol efflux capacity (39), and HDL apolipoprotein A-I (apoA-I) exchange (40, 41). The cell-free fluorometric method utilized in our study to measure HDL_ox_ has been previously applied in several studies of atherosclerotic CVD (31, 36, 42–46). However, these studies had several limitations: (a) They were mostly cross-sectional, (b) had small sample size, (c) had several confounders such as HIV infection, and (d) assessed associations of HDL_ox_ with mostly subclinical atherosclerotic cardiovascular disease and not clinically proven CAD (assessed by cardiac catheterization) and acute coronary events. In a large cohort that assessed the association between serum HDL_ox_ with incident CVD outcomes over 7 years in 139 participants without clinical CVD at baseline who then developed clinical CVD over 7 years of follow-up (CVD group) and 191 healthy control individuals (MASHAD cohort), for every increase in HDL_ox_ by 0.1 unit, there was an increase in CVD risk by 1.62-fold (43). However, the baseline characteristics of the study groups, how these characteristics changed over time, and other major confounders that impact development of CVD were not well described in this study. To our knowledge, no study to date has simultaneously assessed the association of HDL_ox_ with both CVD and ACS to fully characterize the link between HDL_ox_ and the full spectrum of atherosclerotic CVD (both chronic CAD and ACS) in a large study that adjusts for major confounders. In addition, to our knowledge no study to date has shown the feasibility of measuring HDL function in a study of very large sample size in a reproducible, standardized cell-free manner in association with clinical CVD. To address these gaps in knowledge, we determined associations of HDL_ox_ with CAD and ACS in patients with established CVDs in a large cross-sectional observational study. We hypothesize that a reduced antioxidant function of HDL, assessed by HDL_ox_, is not only associated with the presence of coronary atherosclerosis, but also with an acute coronary event.

## Results

### Demographic characteristics of study participants.

A total of 1421 participants were enrolled in this study; 1225 participants were included in the first cohort consisting of elective patients. The second cohort consisted of 196 patients admitted with ACS. Both cohorts were included in the analysis and all 1421 participants were classified according to their coronary status (*n* = 723 in the CAD group, *n* = 502 in the no-CAD group, *n* = 196 with ACS). The characteristics of all study participants are shown in Table 1. The incidence of CAD was higher in participants who were males, with hypertension, diabetes, smokers, and who had hyperlipidemia compared with participants who were females, or with those without the respective risk factors (*P* < 0.001 for all comparisons). Patients suffering from ACS were slightly younger than patients with stable CAD (*P* = 0.031), had less frequently hypertension (*P* = 0.01), and had a lower BMI (*P* = 0.006) compared with the CAD group. Other risk factors like diabetes and smoking did not differ between the groups. The CAD status of the ACS cohort is described in Supplemental Table 1; supplemental material available online with this article; https://doi.org/10.1172/jci.insight.187889DS1

Patients of the ACS group were divided into 2 groups, according to the site of recruitment (*n* = 161 in University Hospital Brandenburg and *n* = 35 in Ruhr-University of Bochum). No differences in the baseline characteristics were found between the 2 groups (all *P* values > 0.05, Supplemental Table 2).

### Patients with CAD had a reduced HDL antioxidant function compared with participants with no CAD.

**Figure 1:**
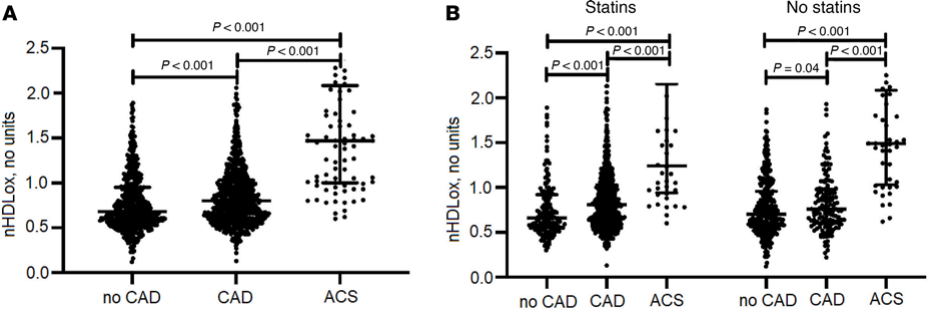
nHDL_ox_ in different study groups and in relation to statins. HDL antioxidant function (nHDL_ox_) was assessed in sera of patients with or without coronary artery disease (CAD) and acute coronary syndrome (ACS) by a biochemical assay as described in the Methods. (**A**) nHDL_ox_ among participants without CAD, with CAD, and with ACS. (**B**) nHDL_ox_ in relation to intake of statins or no intake. The Kruskal-Wallis test was used. If significant, the Mann-Whitney *U* test was used for pairwise comparison. Statistically significant values (*P* < 0.05) are shown in bold numbers.

The 723 patients with CAD had a median of 18% higher nHDL_ox_ (HDL_ox_ normalized by HDL-C levels and the mean value of a pooled serum control from healthy participants) compared with the 502 patients without CAD (*P* < 0.001; Table 1 and Figure 1A). Among all participants with statins (*n* = 755), nHDL_ox_ was higher in participants with CAD compared with participants without CAD (Figure 1B). Patients with CAD on statins had similar levels of nHDL_ox_ compared to patients with CAD not on statins, suggesting that statins did not impact the antioxidant function of HDL in CAD patients. Among CAD participants who were on statins (*n* = 578), males, persons with diabetes, and smokers had higher nHDL_ox_ values compared with females (*P* < 0.001), persons without diabetes (*P* = 0.001), and non-smokers (*P* = 0.002, Supplemental Table 3). Among CAD patients who were not on statins (*n* = 145), males had higher nHDL_ox_ values compared with females (*P* < 0.001 and *P* = 0.002, Supplemental Table 3). Thus, patients with CAD had a reduced HDL antioxidant function compared with participants with no CAD, regardless of statin use.

### Patients with ACS had a reduced HDL antioxidant function compared with participants with CAD.

Patients with ACS had the highest nHDL_ox_ values among all other groups (*P* < 0.001; Table 1 and Figure 1A). An intake of statin prior to the ACS did not influence the level of nHDL_ox_ within the group of patients with ACS in any way (*P* > 0.05). For 148 of the 196 ACS patients, a CAD patient with comparable propensity score could be matched (Table 2). There were 38 female (25.7%) and 110 male (74.3%) pairs. A *P* value of less than 0.001 was calculated for the comparison of the HDL_ox_ values of the ACS patients with the values of the matched CAD patients using Wilcoxon’s signed-rank test.

To minimize confounding factors related to possible slight differences in blood sampling and freezing, we performed a separate analysis based on the center of recruitment (Supplemental Figure 1). The difference between CAD and ACS remained significant, independent of the center of recruitment (*P* < 0.001).

### Associations of nHDL_ox_ levels with serum lipids and instigators of impaired HDL function.

To characterize the clinical relevance of nHDL_ox_ with regard to CVD, we assessed the associations of serum nHDL_ox_ levels with serum lipids and other established mediators of both CAD and HDL functions. These mediators include increased BMI, lipoprotein(a) [Lp(a)] (47), lipoprotein-associated phospholipase A2 (Lp-PLA_2_) (48), glycosylation (hemoglobin A1c; HbA1c) (49), and high-sensitivity C-reactive protein (hsCRP) (1, 14, 17, 36, 39). One hundred ninety-six patients with ACS were not included into this analysis due to a potential bias resulting from the present acute coronary event. However, in all elective study participants (*n* = 1225), males, diabetics, and smokers had higher nHDL_ox_ values compared with females and persons without these risk factors for CVD (*P* < 0.001 each, Supplemental Table 4). In both univariate and multivariate linear regression analyses, among all study participants and also among patients with CVD, male sex, diabetes, higher BMI, LDL, cholesterol, and triglyceride levels were consistently associated with elevated nHDL_ox_ levels (Table 3 and Supplemental Table 5). Associations between nHDL_ox_ and key measures of processes that alter HDL function, such as HDL lipid content (blood triglycerides), glycation (HbA1c), and inflammation (hsCRP) are presented in Supplemental Figure 2. All associations were weak to modest and the strongest association was between HDL_ox_ and triglyceride serum levels across all groups of patients (Table 3 and Supplemental Figure 2).

### Associations of nHDL_ox_ levels with statin use in study participants.

Due to the pleiotropic effects of statins on HDL concentration and function (50) and their established antioxidant effects (51), we analyzed a potential impact of statins on HDL function in CAD by determining the associations of nHDL_ox_ with CVD risk factors in patients with and without statins. The distribution of major risk factors and laboratory parameters based on the intake of statins are presented in Supplemental Table 6. In the univariate and multivariate linear regression analysis, male sex, higher BMI, diabetes, cholesterol, triglycerides, Lp(a), and HbA1c were consistently associated with increased nHDL_ox_ levels in the CAD patient group on statins, as well as when all patient groups were considered together (Supplemental Table 7). Thus, the most consistent correlates of impaired HDL antioxidant function in all participants and in patients with CAD, whether on statins or not, were male sex, increased BMI, and higher triglycerides. It can be assumed that regardless of statin use, male obese patients with dyslipidemia may be at increased risk for reduced HDL antioxidant function, and with that, more at risk for CAD.

### Associations of nHDL_ox_ with CAD.

**Figure 2:**
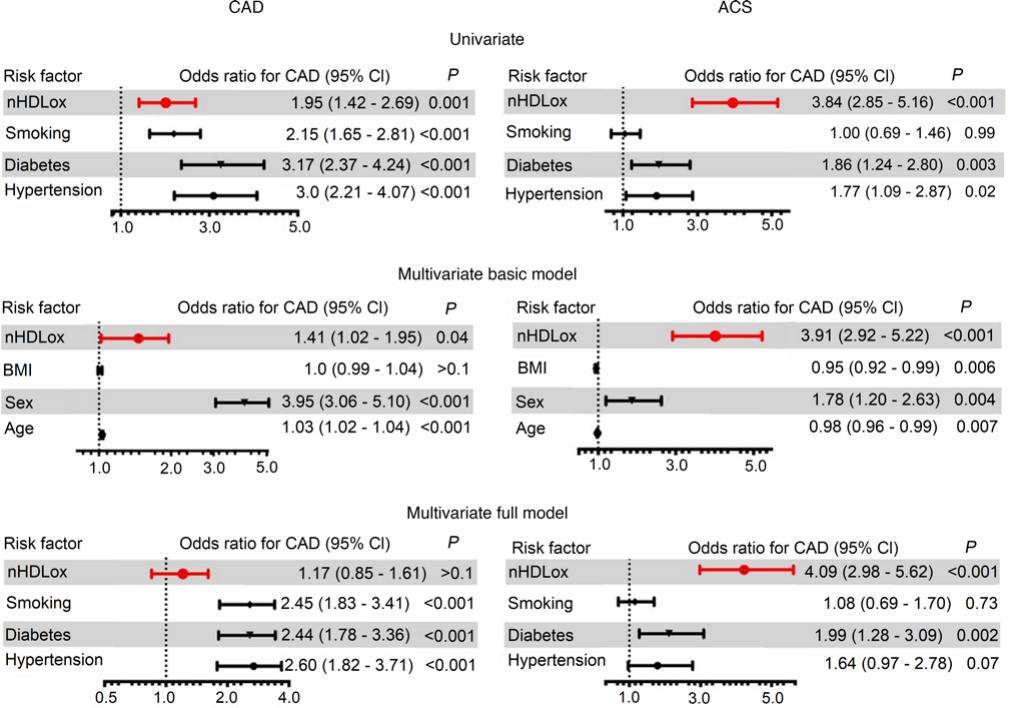
Associations of the antioxidant function of HDL with CAD and ACS. The antioxidant function of HDL (nHDL_ox_) was assessed in sera of patients with or without coronary artery disease (CAD) and acute coronary syndrome (ACS) by a biochemical assay as described in the Methods. The associations of nHDL_ox_ with CAD (versus no CAD) and ACS (versus CAD) were assessed by logistic regression using a univariate model, a basic multivariate model adjusted for age, sex, and body mass index (BMI), and a full multivariate model adjusted for selected risk factors (diabetes, smoking, hypertension, in addition to age, sex, and BMI). ORs and 95%CIs are shown. ORs for continuous variables are per 1-SD increase.

We then determined the associations of nHDL_ox_ levels with CAD. In univariate logistic regression analysis of all study participants, an increase of 1 SD in nHDL_ox_ was associated with 95% increased risk for CAD if compared with participants without CAD (odds ratio [OR] 1.95, 95% confidence interval [95%CI] 1.42–2.69, *P* = 0.001; Figure 2). After adjustment for age, sex, and BMI, nHDL_ox_ remained a significant risk factor for CAD (OR 1.41, 95%CI 1.02–1.95, *P* = 0.04; Figure 2). However, when including other common risk factors in the multivariate analyses, nHDL_ox_ was not significantly associated with CAD (*P* > 0.1, Figure 2). Thus, a reduced serum HDL antioxidant function was not consistently associated with CAD.

### Associations of nHDL_ox_ with ACS.

In the regression analysis, a robust association for nHDL_ox_ as a risk factor for the presence of ACS compared with CAD was seen, independent of traditional risk factors (OR per 1-SD increase in HDL_ox_ = 4.09, 95%CI 2.98–5.62, *P* < 0.001; Figure 2). After adjusting for the recruitment site, the OR remained significant (OR per 1-SD increase in HDL_ox_ = 3.98, 95%CI 2.77–5.41, *P* < 0.001). In summary, increased lipid peroxidation of HDL was strongly associated with the presence of ACS.

### nHDL_ox_ levels in association with the severity of coronary disease in CAD and ACS.

**Figure 3:**
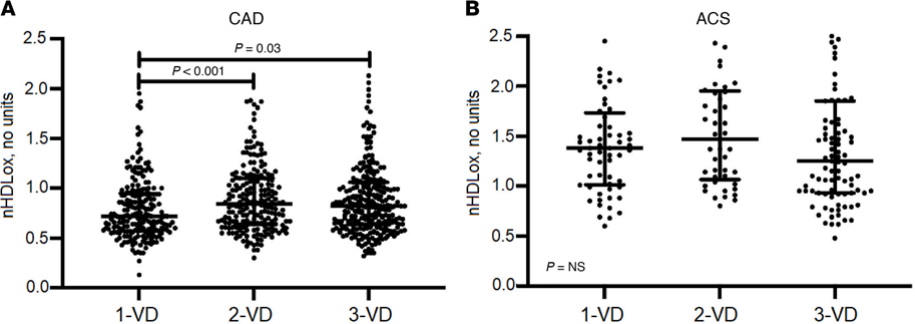
nHDL_ox_ in association with the severity of coronary disease in CAD and ACS. HDL antioxidant function was assessed in sera of patients with CAD (**A**) and ACS (**B**) and categorized based on the number of coronary arteries with a stenosis of 50% or greater in angiography. The Kruskal-Wallis test was used. If significant, the Mann-Whitney *U* test was used for pairwise comparison. Statistically significant values (*P* < 0.05) are shown in bold numbers. 1-VD, 1-vessel disease; 2-VD, 2-vessel disease; 3-VD, 3-vessel disease.

Patients with 1-vessel disease had lower nHDL_ox_ (median 0.72, IQR 0.57–0.95) compared with patients with 2- (median 0.83, IQR 0.64–1.10, *P* < 0.001) and 3-vessel disease (median 0.82, IQR 0.64–1.06, *P* = 0.003; Figure 3A). Among patients with ACS, no statistically significant difference was found by comparing subgroups based on the number of vessels with a stenosis of 50% or greater (*P* > 0.05, Figure 3B).

## Discussion

This study is the largest cross-sectional study to date that explored the association between elevated HDL lipid peroxide (nHDL_ox_) levels, as a measure of decreased HDL antioxidant function, and the occurrence of CAD and ACS. In this study, we found that patients with ACS had a reduced HDL antioxidant function compared with participants with no CAD, regardless of statin use. Increased nHDL_ox_ was strongly associated with acute coronary events, but was inconsistently associated with stable CAD. Increased nHDL_ox_ levels were associated with CAD in univariate analyses of all study participants and patients not taking statins. However, these associations were insignificant in multivariate analyses that accounted for age, sex, BMI, smoking, and hypertension. Patients with 1-vessel disease had lower nHDL_ox_ compared with patients with 2- and 3-vessel disease. The use of propensity score matching showed robust associations of increased nHDL_ox_ levels in patients with ACS compared with patients with CAD. Our results suggest that HDL_ox_ is not only associated with the simple presence of atherosclerosis but also with disease severity and activity, as different levels of HDL_ox_ occur at different stages of CAD. Our study is the largest study published to date (*n* = 1421 patients) that assessed associations of HDL_ox_ with CAD in 2 independent cohorts and specifically with ACS. To our knowledge, the role of HDL_ox_ has not been studied specifically in ACS. Our study also showed the feasibility of measuring HDL function in a study of a very large sample size in a reproducible, standardized cell-free manner in association with clinical CVD.

Our findings support the hypothesis that oxidation of HDL is a strong predictor of atherosclerosis. However, this role seems to be associated with the disease activity; HDL_ox_ is highest in patients with ACS, while patients with stable CAD, on the other hand, still have higher levels than healthy controls. We therefore assume that high HDL_ox_ levels are linked to disease activity or progression rather than reflecting the quantitative atherosclerosis burden of a multivessel CAD with stable plaques. CAD and ACS are 2 different manifestations of the same primary disease, which is atherosclerosis emerging from oxidative stress and vascular inflammation. The presence of CVD risk factors results in oxidative stress and changes in the circulating environment of HDL, leading to increased lipid peroxidation of HDL (HDL_ox_), which results in increased vascular inflammatory activity and CAD development. Although the highest levels of nHDL_ox_ can be found in the presence of an ACS, a definitive causal pathway regarding HDL_ox_ and ACS development still needs to be proven. Acute coronary events fundamentally change the circulating environment for HDL. However, it remains unclear whether highly increased HDL_ox_ levels during ACS contribute to or reflect the pathogenesis of ACS. HDL_ox_ can result from inflammation or oxidation and partially reflect ACS pathogenesis or ongoing damage during myocardial ischemia.

Recently, Yazdandoust et al. demonstrated higher HDL_ox_ levels in patients with angiographically confirmed CAD compared with those without CAD (52). In contrast with our results, the authors also found an association of lipid peroxidation with CAD in the multivariate analysis (52). In the MASAD cohort of 330 individuals who had a median follow-up period of 7 years, plasma baseline HDL_ox_ levels independently predicted risk for clinical CVD, including myocardial infarction, stable angina, unstable angina, or coronary revascularization (43). Higher levels of oxidized HDL have further been correlated with calcium artery score (44) and carotid intima media thickness (36, 53). In a recent study by Lorkowski et al., a low apoA1 exchange rate using a cell-free HDL functional assay was associated with increased incidence of cardiovascular events (54). These observations are supported by the results of further studies, which demonstrated a reduced antiinflammatory, antioxidative HDL function and a reduced cholesterol efflux capacity in patients with ACS or myocardial infarction (55–57).

Consistent with the role of increased systemic inflammation in HDL function and CAD, we found that hsCRP was associated with HDL_ox_ in our study population. This relationship is supported by the findings of Patel et al., who demonstrated a less antiinflammatory capacity of HDL in the ACS setting than controls or individuals with chronic CAD (25). However, our regression analysis found a robust association for HDL_ox_ as a risk factor for the presence of ACS, independent of traditional risk factors also known to be associated with chronic inflammation. This is particularly important, as we have previously demonstrated a significant role of dysfunctional HDL as a driver of HIV-related inflammation, immune dysfunction, and CVD (36, 58). Therefore, it is essential to underline that the role of HDL_ox_ in atherosclerosis may also depend on the context of systemic inflammation and oxidative stress, possibly acting as an additional driver of coronary events in the setting of chronic inflammatory milieu.

Our study also explored the association of increased HDL lipid peroxide content (HDL_ox_) with CVD risk factors that may contribute to developing both CVD and HDL dysfunction. Consistent with the role of oxidative stress and increased glycation in impairment of HDL function, we found that smoking and elevated HbA1c were associated with increased HDL_ox_. The strongest association was seen between HDL_ox_ and triglyceride serum levels, a marker of insulin resistance (59), in accordance with prior evidence that triglyceride content of HDL is associated with HDL dysfunction (60). Similar findings on the association of HDL_ox_ with risk factors were recently shown in a large national cohort of Americans (61). Furthermore, as our analysis included age as a matching criterion, we observed these differences of nHDL_ox_ levels consistently in all matched pairs. Thus higher nHDL_ox_ levels affect all age groups of patients presenting with ACS, despite prior evidence of an age-dependent increased overall oxidation status (62). HDL-C levels in our study were also significantly lower in the ACS group compared with the CAD or no-CAD group. This finding is in line with results from previous research that frequently found lower HDL-C in patients with acute coronary events (63, 64).

We showed that a validated cell-free biochemical assay that determines reduced HDL antioxidant function is a scalable tool for large-scale studies to investigate the role of HDL function in the development of atherosclerosis in vivo. The cell-free fluorometric method utilized in our study to measure HDL_ox_ has been previously applied in several studies of atherosclerotic CVD (31, 36, 42–46). However, these studies were mostly cross-sectional, with a small sample size, and assessed associations of HDL_ox_ with mostly subclinical atherosclerotic CVD. Our data show clear associations between a decreased HDL antioxidant function and the occurrence of CAD and ACS in a well-characterized, large cohort. It is further known that results from this assay show strong correlations with previously validated cell-based and cell-free assays of HDL function, including antiinflammatory function of HDL (31, 36), cholesterol efflux capacity (39), and HDL apoA-I exchange (40, 41). Consequently, as lipid peroxidation plays an important role in the inflammation of artery walls (1, 16, 65), HDL redox properties can be used as a potential marker of CVD and biological processes in humans (36, 43). One example for a potential use would be the validation of the Framingham risk score with HDL_ox_ instead of HDL-C (66) to improve CVD risk prediction. Therefore, quantifying nHDL_ox_ is feasible as a reproducible and scalable test to study HDL antioxidant function.

PEG precipitates from serum contain HDL and several other plasma proteins (67, 68). Therefore, the oxidation status cannot be attributed exclusively to HDL. Although PEG precipitation is a reproducible, high-throughput method to extract HDL from patient serum, a significant issue with this method is the contamination of the HDL with other plasma proteins, especially albumin. We have previously determined the impact of proteins in the PEG fraction on the Amplex Red assay of HDL_ox_ (36). Using immunoaffinity capture of HDL and 2 different methods to detect albumin content, we showed that the HDL captured on 96-well plates is largely free of albumin. Using this method with different matrices (HDL isolation by ultracentrifugation vs. PEG method vs. immunoaffinity capture), we demonstrated that HDL from patients with dysfunctional HDL has a higher rate of lipid peroxidation of a specific amount of HDL compared with HDL from healthy patients. Similar results in the readout were observed when HDL-C was isolated by FPLC or ultracentrifugation or PEG. Our results in combination with the biochemical principle of the assay (HRP-dependent detection of Amplex Red is linked to the lipid peroxide content of HDL and not protein oxidation) suggest that although glycated proteins and protein peroxides are important determinants of HDL function (69), they are not important drivers of the HDL_ox_ readout of our assay.

The effect of apoB depletion on measurements of HDL function has been previously evaluated (70). We have previously validated the cell-free fluorometric method of HDL function using apoB-depleted serum and ultracentrifugation with purified HDL (31, 36, 71). The relative differences in measures of HDL function among samples isolated with an identical method of HDL isolation will not be significantly affected in a given study. HDL isolation by PEG precipitation used for this assay is a high-throughput method and can be scaled up in a large study like ours (>1000 participants). Additional studies of HDL_ox_ in association with CAD using HDL isolated by ultracentrifugation are needed to further validate our findings.

Our study has limitations. The absence of the association of HDL_ox_ with clinical CAD in multivariate analyses of our study participants may be related to our cross-sectional study design. A limitation of our study was the lack of data regarding associations of HDL_ox_ with adverse cardiovascular clinical outcomes. However, our study gives indirect insight into the association of HDL_ox_ with adverse CVD clinical outcomes since CAD and ACS are established correlates of adverse CVD clinical outcomes. Notably, the simultaneous assessment of HDL_ox_ in association with different spectrums of atherosclerotic CVD (from stable CAD to ACS) provides useful insight into the role of HDL_ox_ in atherosclerotic CVD, especially considering our prior mechanistic data showing that HDL_ox_ drives ex vivo atherogenesis in a mechanistic model of atherogenesis (36). Finally, the potential for reverse causality in the relationship between HDL_ox_ and acute coronary events is a potential limitation of our study. While our findings suggest a significant association between elevated levels of HDL_ox_ and the incidence of ACS, it is important to consider that this relationship may not be purely causal. Reverse causality implies that rather than HDL_ox_ contributing to the development of ACS, the presence of ACS itself could lead to increased levels of HDL_ox_. Our observational study does not prove causality and may be subject to unknown confounders, such as the use of various medications that may impact HDL function. We assessed serum levels of HDL_ox_ at only one point of time. The development and progression of atherosclerosis is a dynamic, complex process, and serial assessments of specific instigators of atherosclerosis may be needed to fully capture the role of these mediators of atherogenesis in the development of clinical CAD. To address these limitations, future experimental studies investigating the mechanistic pathways linking HDL_ox_ to ACS could help clarify whether oxidized HDL plays a causal role in the development of acute coronary events.

In conclusion, this is the largest cross-sectional cohort study to quantify HDL lipid peroxide content (HDL_ox_) as a routine serum test to assess HDL antioxidant function using what we believe is a novel, previously validated fluorometric biochemical cell-free assay. We provide the first evidence to our knowledge of an association between HDL_ox_ and the severity of CAD; patients with ACS had higher serum concentrations of HDL_ox_ compared with those with stable CAD, independent of the presence of classical CVD risk factors. Furthermore, patients with stable CAD showed higher HDL_ox_ concentrations than healthy participants. Our data support the thesis that reduced HDL antioxidant function significantly contributes to the progression of subclinical atherosclerosis to manifested CAD. New therapeutic approaches to improve the reduced efflux capacity after myocardial infarction are now under investigation (72). Furthermore, longitudinal studies are required to investigate a possible causal role of HDL_ox_ in the setting of acute and chronic CVDs and establish the role of HDL function in CVD risk prediction.

## Methods

### Sex as a biological variable.

Our study examined male and female humans; similar findings are reported for both sexes.

### Study design.

The study was conducted at the University hospital of Brandenburg and at the University hospital of Bochum. We enrolled 2 independent cohorts. The first cohort consisted of patients admitted for elective coronary angiography between 2017 and 2019. Inclusion criteria were known or suspected diagnosis of CAD with or without risk factors for CVD and an indication for elective coronary angiography independent of this study. Exclusion criteria were known cancer disease, acute infectious disease, known rheumatic disease, and age less than 18 years.

The second cohort consisted of patients with ACS who underwent urgent coronary angiography between 2019 and 2021. Patients with ACS met the same exclusion criteria as the elective patients and were enrolled consecutively. Only patients with type 1 myocardial infarction were included in the analysis. Patients testing positive for COVID-19 were excluded from analysis. Based on the findings of angiography, patients who had an at least 50% stenosis in the diameter of a coronary artery were assigned to the CAD group. Patients with no stenosis or stenosis of less than 50% in the diameter of all coronary arteries were assigned to the no-CAD group.

### Biomarker and laboratory assessment.

Blood sampling was performed after overnight fasting before any procedures in the elective patients or within 48 hours after admission for ACS (mostly after coronary angiography). Serum was prepared from blood samples and was cryopreserved (–80°C). Grossly hemolyzed serum was excluded from further analysis.

Standard clinical assays were conducted within the central laboratory unit of the university hospital–obtained laboratory parameters. hsCRP was measured using the Tina-Quant CRP (Latex) kit (Roche Diagnostics). Lp-PLA_2_ was determined by Diasys Lp-PLA2 activity reagent (Diasys Diagnostic Systems), according to the manufacturer’s instructions.

### Assessment of HDL_ox_.

HDL_ox_ was quantified in serum using a previously validated fluorometric biochemical cell-free assay that measures HDL lipid peroxide content based on the oxidation of the fluorochrome Amplex Red (36, 38). First, serum was depleted of apoB by PEG precipitation. Fifty microliters of apoB-depleted serum was then added to wells of a 96-well plate in duplicate followed by addition of 0.075 units per well of HRP and 50 μM Amplex Red reagent for a total volume of 100 μL. HRP catalyses the reaction of Amplex Red to resorufin in combination with endogenous peroxides. After 1 hour, the fluorescence of resorufin at wavelengths of 535/590 nm was determined using a Spark 10M microplate reader (Tecan). To standardize the assay and minimize experimental variability, apoB-depleted sera from 10 healthy volunteers (not study participants) were pooled and were used as experimental control in each plate. Mean fluorescence from each sample was normalized by the mean fluorescence readout of the pooled control and HDL-C using the following calculation: nHDL_ox_ = (HDL_ox_ sample × 47 [mg/dL])/(HDL_ox_ control × HDL-C sample [mg/dL]), where 47 mg/dL represents HDL-C of the pooled serum control. Samples were analyzed after recruitment of the last patient. The intra-assay coefficient of variation (CV) was 6.7%. The inter-assay CV was 3.7%. All laboratory measurements were performed in Brandenburg, Germany.

### Statistics.

The distribution of data was checked using the Shapiro-Wilk test. If the assumption of normal distribution was not rejected (*P* > 0.1), then the comparison of the 2 groups was performed with a *t* test (>2 groups: 1-way ANOVA). If the normal distribution assumption was rejected, the Mann-Whitney *U* test was applied (>2 groups: Kruskal-Wallis test). Values are given as median with IQR. The χ^2^ test was used to compare the frequency of a categorical variable between independent groups. Pearson’s correlation coefficient was assessed for correlations between 2 continuous variables with normal distribution based on the Shapiro-Wilk test. To further assess differences in HDL_ox_ levels between CAD and ACS patients, a propensity score matching analysis was performed applying the following matching criteria: sex, age, HDL, and BMI (matching method: nearest neighbor, caliper width = 0.25, 1:1 matching, no replacement). Distribution of the matching criteria in the 2 groups was checked using Wilcoxon’s signed-rank test. Univariate logistic regression analysis was used to assess the relationship between continuous and categorical variables with the presence of disease (CAD or ACS vs. no disease). Univariate linear regression analysis was used to assess the relationship between continuous and categorical variables with the continuous variable nHDL_ox_. Multivariate analyses (logistic regression) were performed adjusted for age, sex, and BMI in a base model and in addition for diabetes mellitus, hypertension, and presence of CAD in the full model. All tests were 2-tailed. A *P* value of less than 0.05 was considered significant. The statistical analyses were performed by an experienced statistician using SAS/STAT and GraphPad Prism software.

### Study approval.

Written informed consent was obtained from all participants. The study was approved by the local ethics committees of the medical association of Brandenburg (no. AS69bB/2016) and of the Ruhr-University of Bochum (no. 15-5279) in accordance with the Declaration of Helsinki.

### Data availability.

The data that support the findings of this study are available in the Supporting Data Values file or from the corresponding author upon reasonable request.

## Author contributions

NP, BS, and TK conceptualized and designed the study, provided overall management and supervision, analyzed and interpreted data, and led in manuscript drafting. RM, LS, and MJ collected and processed the blood samples, obtained HDL_ox_ measurements, and acquired and curated data. WD, FSS, PH, and IB contributed to the implementation of the research, and to the analysis and the interpretation of the work. NH, OR, THW, MC, and CU made substantial contributions to study conceptualization and design, revising the work critically for important intellectual content, and validated the work. NP, BS, TK, and OR obtained funding. All authors discussed the results and reviewed the manuscript.

## Supplementary Material

Supplemental data

ICMJE disclosure forms

Supporting data values

## Figures and Tables

**Table 1:**
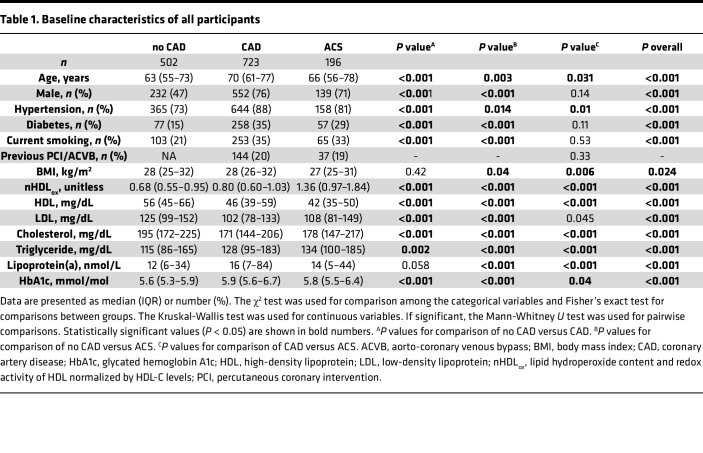
Baseline characteristics of all participants

**Table 2:**
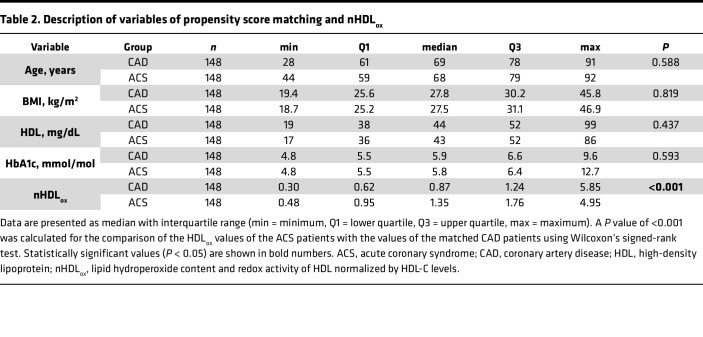
Description of variables of propensity score matching and nHDL_ox_

**Table 3:**
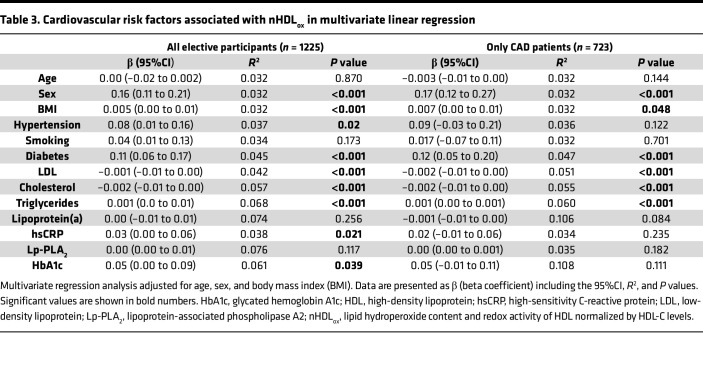
Cardiovascular risk factors associated with nHDL_ox_ in multivariate linear regression

## Version 1 03/24/2025

Electronic publication
