# Supplementary material for: Reduced high-density lipoprotein antioxidant function in patients with coronary artery disease and acute coronary syndrome
Source: JCI Insight. 2025 Mar 24;10(6):e187889. doi: 10.1172/jci.insight.187889 (PMC11949010; doi:10.1172/jci.insight.187889)
Supplement: Supplemental data [file jciinsight-10-187889-s239.pdf]

Supplemental Table 1. Distribution of CAD

CAD	Frequency	Percent	Valid percent	Cumulative percent
**1-vessel**	60	30.6	30.6	30.6
**2-vessel**	45	23.0	23.0	53.6
**3-vessel**	91	46.4	46.4	100.0
**Total**	196	100.0	100.0	

Data are presented as frequencies in numbers and percentages. CAD: coronary artery disease

Supplemental Table 2. Baseline characteristics of ACS patients based on the site of recruitment

	University Hospital Brandenburg	Ruhr University Bochum	p-value
**N**	**161**	**35**	
**Age, years**	65 (55-77)	68 (59-80)	0.200
**Male, no (%)**	114 (71)	25 (71)	1.000
**Hypertension, no (%)**	129 (80)	29 (83)	1.000
**Diabetes, no (%)**	43 (27)	14 (40)	0.151
**Current smoking, no (%)**	55 (34)	10 (29)	0.424
**Previous PCI/ACVB, no (%)**	26 (16)	8 (22)	0.056
**BMI, kg/m^2**	27 (25-31)	29 (24-33)	0.475
**nHDL$_{ox}$, no unit**	1.32 (0.99-1.75)	1.51 (0.89-2.02)	0.414
**HDL, mg/dl**	42 (34-50)	43 (35-54)	0.659
**LDL, mg/dl**	106 (80-151)	109 (84-144)	0.854
**Cholesterol, mg/dl**	178 (150-220)	176 (147-197)	0.517
**Triglyceride, mg/dl**	140 (104-196)	121 (87-170)	0.061
**Lipoprotein a, nmol/l**	28 (11-107)	48 (19-104)	0.216
**HbA1c, mmol/mol**	5.8 (5.5-6.3)	5.9 (5.6-6.5)	0.220

Data are presented as median with interquartile range or number (%). Chi square test was used for comparison among the categorical variables and Fisher exact test for comparisons between groups. The Kruskal-Wallis-Test was used for continuous variables. If significant, the Mann-Whitney U Test was used for pairwise comparisons. Statistically significant values ($p<0.05$) are shown in bold numbers. p-value[1]: comparison no CAD versus CAD. p-value[2]: comparison no CAD versus ACS. p-value[3]: comparison CAD versus ACS. Abbreviations: ACVB: aorto-coronary venous bypass,

BMI: body mass index, CAD: coronary artery disease, HbA1c: glycated hemoglobin A1c, HDL: high-density lipoprotein, LDL: low-density lipoprotein, $nHDL_{ox}$: lipid hydroperoxide content and redox activity of HDL normalized by HDL-C levels, no: number, PCI: percutaneous coronary intervention.

Supplemental Table 3. $nHDL_{ox}$ levels with regards to main risk factors and intake of statins in participants with coronary artery disease

	Statins (n=578)		No statins (n=145)	
	**$nHDL_{ox}$**	**p-value**	**$nHDL_{ox}$**	**p-value**
Males, no (%)	0.83 (0.65-1.07)	< **0.001**	0.83 (0.63-1.07)	**0.002**
Females, no (%)	0.66 (0.55-0.93)		0.68 (0.54-0.83)	
Diabetes, no (%)	0.86 (0.65-1.15)	**0.001**	0.81 (0.64-1.149	0.12
No Diabetes, no (%)	0.76 (0.60-0.98)		0.74 (0.59-0.97)	
Current smoking, no (%)	0.86 (0.66-1.1)	**0.002**	0.81 (0.62-1.06)	0.28
No current smoking	0.78 (0.59-1.0)		0.73 (0.60-0.97)	
Hyperlipidaemia	0.80 (0.60-1.05)	0.58	0.74 (0.58-0.98)	0.49
No Hyperlipidaemia	0.81 (0.65-1.0)		0.77 (0.62-1.0)	

Data are presented as median with interquartile range. Mann-Whitney U Test were performed. Significant values are shown in bold numbers. Missing information about statins in 4 participants. $nHDL_{ox}$: lipid hydroperoxide content and redox activity of HDL normalized by HDL-C levels.

Supplemental Table 4. $nHDL_{ox}$ with regards to main risk factors for coronary artery disease

	**nHDLox**	**p-value**
Males	0.79 (0.62-1.06)	**< 0.001**
Females	0.66 (0.53-0.88)	
Diabetes	0.82 (0.62-1.12)	**< 0.001**
No Diabetes	0.72 (0.57-0.97)	
Current smoking	0.81 (0.62-1.06)	**< 0.001**
No current smoking	0.71 (0.57-0.98)	
Hypertension	0.75 (0.59-1.0)	0.07
No Hypertension	0.71 (0.55-0.99)	

Data are presented as median with interquartile range. Mann-Whitney U Test were performed. Significant values are shown in bold numbers. nHDLox: lipid hydroperoxide content and redox activity of HDL normalized by HDL-C levels.

Supplemental Table 5. Cardiovascular risk factors associated with $nHDL_{ox}$ in univariate linear regression

	All elective participants (no ACS)			Only CAD patients		
	β (95% CI)	R^2	p-value	β (95% CI)	R^2	p-value
**Age**	0.00 (-0.01 to 0.01)	0.000	0.707	-0.004 (-0.01 to 0.01)	0.007	**0.028**
**Sex**	0.157 (0.11 to 0.21)	0.029	**<0.001**	0.169 (0.09 to 0.25)	0.022	**< 0.001**
**BMI**	0.005 (0.00 to 0.01)	0.003	**0.05**	0.008 (0.00 to 0.02)	0.006	**0.047**
**Hypertension**	0.08 (0.02 to 0.14)	0.005	**0.015**	0.058 (-0.06 to 0.17)	0.001	0.316
**Smoking**	0.063 (0.01 to 0.12)	0.004	**0.024**	0.052 (-0.02 to 0.13)	0.003	0.169
**Diabetes**	0.131 (0.08 to 0.19)	0.018	**<0.001**	0.119 (0.05 to 0.19)	0.014	**0.002**
**LDL**	-0.002 (-0.01 to 0.0)	0.020	**<0.001**	-0.002 (-0.01 to 0.00)	0.026	**< 0.001**
**Cholesterol**	-0.002 (-0.01 to 0.0)	0.041	**<0.001**	-0.002 (-0.01 to 0.00)	0.033	**< 0.001**
**Triglycerides**	0.001 (0.00 to 0.01)	0.050	**<0.001**	0.001 (0.00 to 0.01)	0.041	**< 0.001**
**Lipoprotein a**	0.00 (-0.01 to 0.00)	0.002	0.278	-0.001 (-0.01 to 0.01)	0.007	0.138
**hsCRP**	0.029 (0.01 to 0.05)	0.006	**0.017**	0.019 (-0.01 to 0.05)	0.002	0.218
**Lp-PLA2**	0.00 (0.00 to 0.01)	0.007	**0.007**	0.00 (0.00 to 0.01)	0.006	**0.044**
**HbA1c**	0.066 (0.02 to 0.11)	0.015	**0.003**	0.064 (0.00 to 0.13)	0.013	**0.043**

Data are presented as β (beta coefficient) incl. 95% CI (confidence interval), R^2 and p value. Significant values are shown in bold numbers. Abbreviations: HbA1c: glycated hemoglobin A1c, HDL: high-density lipoprotein, hsCRP: high sensitive C reactive protein, LDL: low-density lipoprotein, Lp-PLA2: lipoprotein associated phospholipase A2, $nHDL_{ox}$: lipid hydroperoxide content and redox activity of HDL normalized by HDL-C levels.

Supplemental Table 6. Baseline characteristics of participants with coronary artery disease with and without statins

	Statins	No statins	p-value
**N**	**578**	**145**	
**Age, years**	70 (61-77)	67 (59-76)	**0.04**
**Male, no (%)**	451 (78)	101 (70)	**0.04**
**Hypertension, no (%)**	521 (90)	123 (85)	0.07
**Diabetes, no (%)**	210 (36)	48 (33)	0.49
**Current smoking, no (%)**	201 (35)	52 (36)	0.92
**Previous PCI/ACVB, no (%)**	125 (22)	19 (13)	0.05
**BMI, kg/m^2**	28 (26-32)	28 (25-32)	0.27
**nHDLox, no unit**	0.81 (0.61-1.03)	0.76 (0.6-0.98)	0.29
**HDL, mg/dl**	46 (39-58)	51 (40-65)	**0.007**
**LDL, mg/dl**	95 (75-121)	137 (104-1639	**<0.001**
**Cholesterol, mg/dl**	166 (141-193)	207 (174-235)	**<0.001**
**Triglyceride, mg/dl**	128 (94-185)	127 (102-181)	0.66
**Lipoprotein a, mmol/l**	16 (7-84)	14 (6-91)	0.77
**hsCRP, mg/dl**	0.09 (0.00-0.59)	0.2 (0.00-0.79)	0.09
**Lp-PLA$_2$, U/L**	345 (288-422)	430 (358-528)	**<0.001**
**HbA1c, mmol/mol**	5.9 (5.6-6.8)	5.8 (5.5-6.7)	0.65

Data are presented as median with interquartile range or number (%). Chi square test for categorical variables and Mann-Whitney U Test for metric variables were performed. Significant values are shown in bold numbers. NA denotes not applicable. ACVB aorto-coronary venous bypass, BMI body mass index, CAD coronary artery disease, HbA1c glycated hemoglobin A1c, HDL high-density lipoprotein, hsCRP high sensitive C reactive protein, LDL low-density lipoprotein, Lp-PLA2 lipoprotein associated phospholipase A2, PCI percutaneous coronary intervention, nHDL$_{ox}$: lipid hydroperoxide content and redox activity of HDL normalized by HDL-C levels.

Supplemental Table 7. Cardiovascular risk factors associated with $nHDL_{ox}$ in univariate and multivariate linear regression

	CAD on statins (n=578)					
	Univariate			Multivariate		
	**β (95% CI)**	**R^2**	**p-value**	**β (95% CI)**	**R^2**	**p-value**
**Age**	-0.005 (-0.009 to -0.001)	0.009	**0.022**	-0.004 (-0.008 to 0.000)	0.027	0.068
**Sex**	0.149 (0.05 to 0.249)	0.015	**0.003**	0.151 (0.047 to 0.256)	0.027	**0.005**
**BMI**	0.007 (-0.002 to 0.016)	0.005	0.11	0.008 (-0.001 to 0.016)	0.027	0.098
**Hypertension**	0.044 (-0.095 to 0.183)	0.001	0.531	0.09 (-0.056 to 0.236)	0.030	0.226
**Smoking**	0.05 (-0.038 to 0.137)	0.002	0.263	0.008 (-0.095 to 0.112)	0.026	0.874
**Diabetes**	0.12 (0.034 to 0.205)	0.013	**0.006**	0.134 (0.044 to 0.223)	0.042	**0.003**
**LDL**	-0.002 (-0.003 to -0.001)	0.018	**0.001**	-0.002 (-0.003 to -0.001)	0.040	**0.002**
**Cholesterol**	-0.002 (-0.003 to -0.001)	0.027	**<0.001**	-0.002 (-0.003 to -0.001)	0.048	**< 0.001**
**Triglycerides**	0.001 (0.00 to 0.001)	0.028	**<0.001**	0.001 (0.00 to 0.001)	0.043	**< 0.001**
**Lipoprotein a**	-0.001 (-0.001 to 0.00)	0.006	0.211	-0.001 (-0.001 to 0.00)	0.100	0.180
**hsCRP**	0.008 (-0.026 to 0.042)	0.000	0.637	0.011 (-0.029 to 0.051)	0.031	0.586
**Lp-PLA2**	0.00 (0.00 to 0.001)	0.008	**0.034**	0.00 (0.00 to 0.001)	0.035	0.138
**HbA1c**	0.049 (-0.024 to 0.123)	0.007	0.187	0.043 (-0.029 to 0.116)	0.086	0.241

Multivariate regression analysis adjusted for age, gender and body mass index (BMI). Data are presented as β (beta coefficient) incl. 95% CI (confidence interval), R^2 and p-value. Significant values are shown in bold numbers. Abbreviations: HbA1c: glycated hemoglobin A1c, HDL: high-density lipoprotein, hsCRP: high sensitive C reactive protein, LDL: low-density lipoprotein, $Lp\text{-}PLA_2$: lipoprotein associated phospholipase A_2, $nHDL_{ox}$: lipid hydroperoxide content and redox activity of HDL normalized by HDL-C levels.

Supplemental Figure 1. Levels of nHDL$_{ox}$ based on the center of recruitment

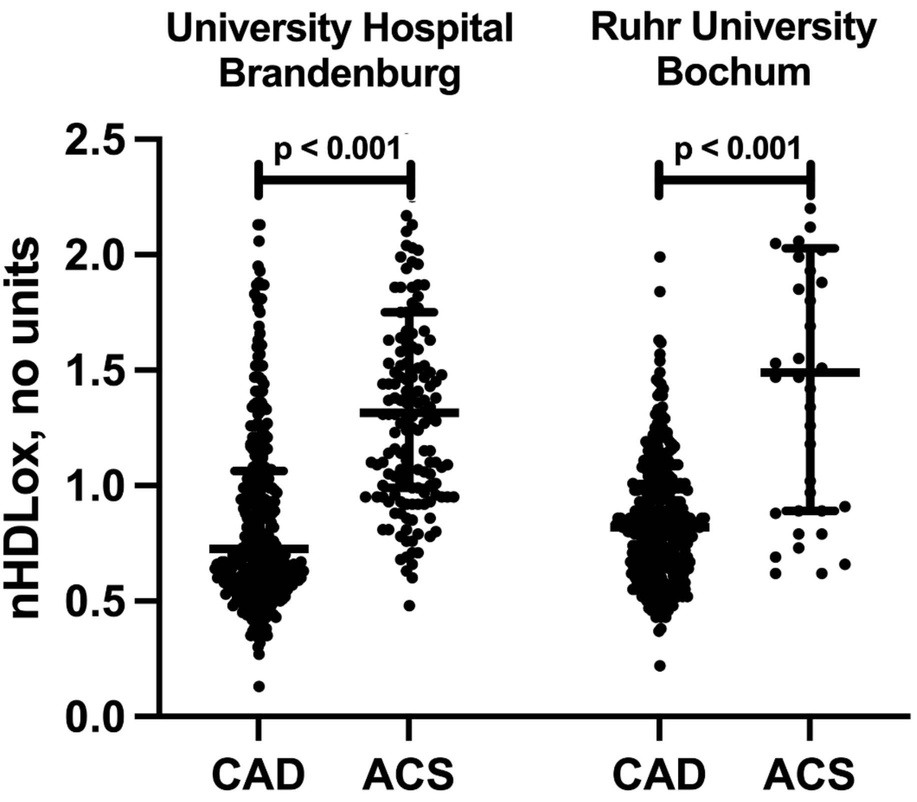

Additional analysis of nHDL$_{ox}$ levels based on the center of recruitment. Data are presented as median with interquartile range (IQR). The Kruskas-Wallis-Test was used. If significant, the Mann-Whitney U Test was applied to compare groups. Statistically significant values (p<0.05) are shown in bold numbers. ACS: acute coronary syndrome, CAD: coronary artery disease, nHDL$_{ox}$: lipid hydroperoxide content and redox activity of HDL normalized by HDL-C levels.

Supplemental Figure 2. Correlations of $nHDL_{ox}$ with triglycerides, HbA1c and hsCRP

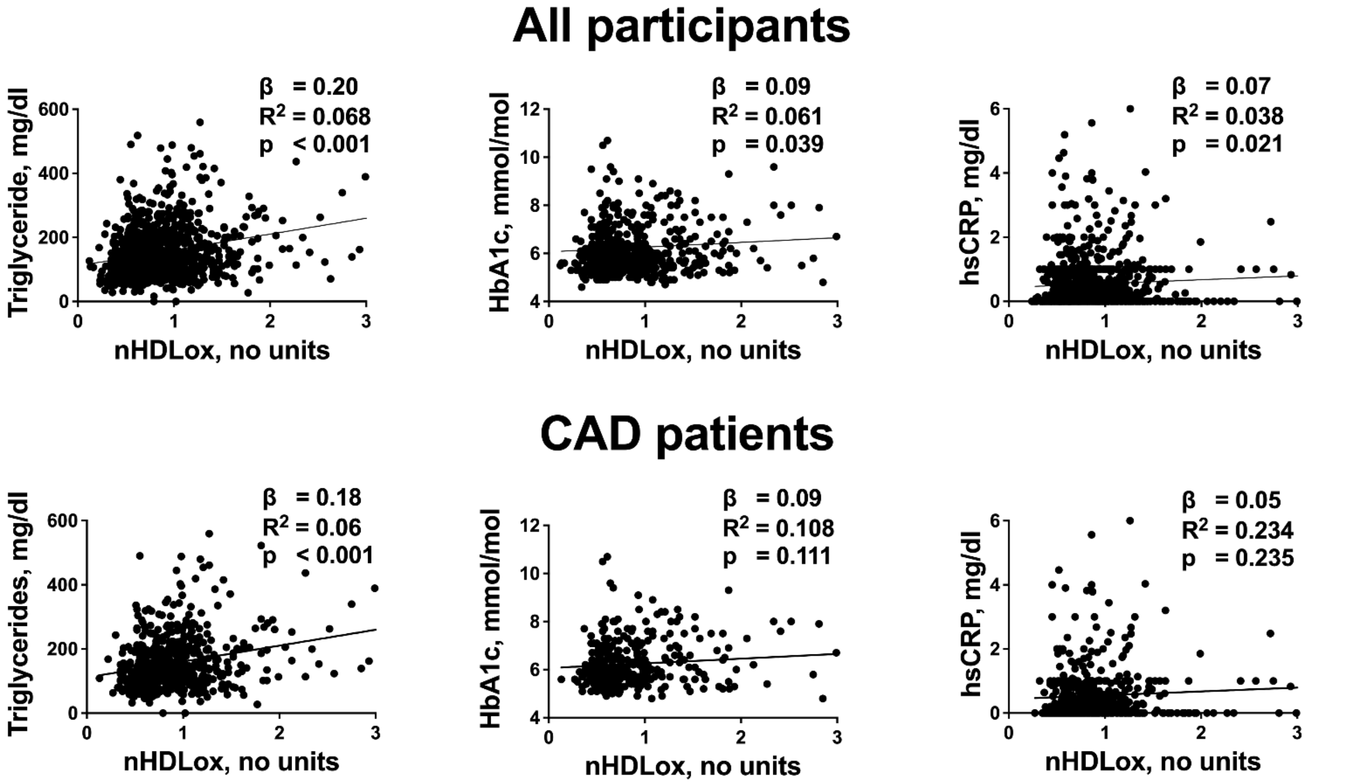

Multivariate regression analysis adjusted for age, gender and body mass index (BMI). Only elective participants are analyzed (no acute coronary syndrome). Statistically significant values ($p<0.05$), β coefficient and R^2 are shown in bold numbers. CAD: coronary artery disease, HbA1c: glycated hemoglobin A1c, hsCRP: high sensitive C reactive protein, $nHDL_{ox}$: lipid hydroperoxide content and redox activity of HDL normalized by HDL-C levels.
